# Inflammation Promotes a Conversion of Astrocytes into Neural Progenitor Cells via NF-κB Activation

**DOI:** 10.1007/s12035-015-9428-3

**Published:** 2015-09-17

**Authors:** Sebastien Gabel, Eric Koncina, Gauthier Dorban, Tony Heurtaux, Cindy Birck, Enrico Glaab, Alessandro Michelucci, Paul Heuschling, Luc Grandbarbe

**Affiliations:** 1 Life Sciences Research Unit, Faculty of Science, Technology and Communication, University of Luxembourg, Campus Limpertsberg, 162A, avenue de la Faïencerie, L-1511 Luxembourg, Luxembourg; 2 Luxembourg Centre for Systems Biomedicine, University of Luxembourg, Campus Belval, 7, avenue des Hauts-Fourneaux, Esch-Belval, G.D., L-4362 Esch-sur-Alzette, Luxembourg

**Keywords:** Astrocyte, Inflammation, NF-κB, Dedifferentiation, Neural progenitor cells

## Abstract

Brain inflammation, a common feature in neurodegenerative diseases, is a complex series of events, which can be detrimental and even lead to neuronal death. Nonetheless, several studies suggest that inflammatory signals are also positively influencing neural cell proliferation, survival, migration, and differentiation. Recently, correlative studies suggested that astrocytes are able to dedifferentiate upon injury and may thereby re-acquire neural stem cell (NSC) potential. However, the mechanism underlying this dedifferentiation process upon injury remains unclear. Here, we report that during the early response of reactive gliosis, inflammation induces a conversion of mature astrocytes into neural progenitors. A TNF treatment induces the decrease of specific astrocyte markers, such as glial fibrillary acidic protein (GFAP) or genes related to glycogen metabolism, while a subset of these cells re-expresses immaturity markers, such as CD44, Musashi-1, and Oct4. Thus, TNF treatment results in the appearance of cells that exhibit a neural progenitor phenotype and are able to proliferate and differentiate into neurons and/or astrocytes. This dedifferentiation process is maintained as long as TNF is present in the culture medium. In addition, we highlight a role for Oct4 in this process, since the TNF-induced dedifferentiation can be prevented by inhibiting Oct4 expression. Our results show that activation of the NF-κB pathway through TNF plays an important role in the dedifferentiation of astrocytes via the re-expression of Oct4. These findings indicate that the first step of reactive gliosis is in fact a dedifferentiation process of resident astrocytes mediated by the NF-κB pathway.

**Electronic supplementary material:**

The online version of this article (doi:10.1007/s12035-015-9428-3) contains supplementary material, which is available to authorized users.

## Introduction

The most widely recognized form of astrocyte plasticity is the one which is acquired after injury or during disease states in the central nervous system (CNS). At sites of ongoing CNS inflammation, local populations of astrocytes become hypertrophic, undergo changes in gene expression, and proliferate (reviewed in [1–3]). Although the regulation of astrocyte differentiation during CNS development is largely described [4, 5], the molecular mechanisms controlling the proliferation of [6] reactive astrocytes and the formation of the glial scar after pathological insults are not yet fully understood. Because a subset of these reactive astrocytes also divides, it has been suggested that these cells arise from endogenous progenitors present in the adult brain [7]. Interestingly, a potential dedifferentiation of astrocytes has already been suggested previously [8]. Recent reports have shown that some astrocytes are able to acquire stem cell properties upon injury and hence may provide a promising cell type to initiate repair [9, 10]. In addition, recent in vitro studies have suggested that mature astrocytes could recover the potential of neural stem cells [11, 12]. These different findings suggest that in the first step of reactive gliosis, some mature astrocytes undergo morphological changes, regain the capacity to proliferate, and become multipotent progenitor cells, characterized by the expression of typical stemness markers. In the CNS, neural progenitors express stemness markers, such as Nestin, Sox2, CD44, Musashi-1, and Oct4 [13, 14]. It has been suggested that CD44-positive cells represent astrocyte precursor cells, but neural stem cells as well as other types of precursor cells do also express CD44 [15]. Oct4 expression is believed to be restricted to pluripotent cells [16, 17]. Nevertheless, some reports show that Oct4 is present in neurospheres, which are composed of neural stem cells and more restricted neural progenitor cells, and that up- or downregulation of Oct4 influences cell fate and differentiation [18, 19]. In the same way, Oct4 is strongly expressed in glioma and the expression level can be positively correlated to increasing glioma grades [20].

Our study shows that inflammation induces a switch of astrocytes into neural progenitors. In a first step, TNF-activated astrocytes lose the expression of glial fibrillary acidic protein (GFAP) and genes related to glycogen metabolism. Simultaneously, they re-express stemness markers, such as CD44, Musashi-1, and Oct4. This re-expression of immaturity markers is maintained as long as TNF is present in the culture medium. In addition, we provide a role for Oct4 in this process of dedifferentiation. The dedifferentiation induced by TNF can be prevented by decreasing the expression of Oct4. Our results show that Oct4 can, at least partially, control this switch. Thus, the activation of the NF-κB pathway appears to be a key element in the dedifferentiation process of astrocytes, which seems to represent the first step characterizing the reactive gliosis.

## Materials and Methods

### Ethics Statement

The experiments involving animals were carried out according to the 2010/63 European Union Directive and internal ethical committee regulations.

### Animals

Wild-type male C57Bl/6J mice (Harlan, The Netherlands) were housed in an air-conditioned room with free access to water and food, using standard guidelines. Mice were used at 6–8 weeks of age. The mice were randomly dispatched into six groups of eight mice. In every group, three animals were used for RNA extractions and three animals for immunohistochemistry analysis. The animal experiments were carried out in strict accordance with our local Committee for Care and Use of Laboratory Animals.

### Stab Wound Injury and Immunohistofluorescence

The animals were anesthetized with isoflurane and mounted into a stereotaxic frame (David Kopf Instruments, Tujunga, CA, USA). Stab wound injury was executed with phosphate-buffered saline (PBS) or LPS (1 μg/μl) or TNF (10 ng/μl) in the right primary somatosensory cortex (S1 of C57BL/6J mice). The wound was inflicted using a Hamilton syringe (7001 SN 1 μl) left in place during 5 min at the following stereotaxic coordinates: 2 mm anterior, 2.3 mm lateral, and 2.5 mm ventral to the bregma. For the kinetic experiments, the wound was inflicted at the following stereotaxic coordinates: −1.1 mm anterior, 2.5 mm lateral, and 1.1 mm ventral to the bregma. Twenty-four hours, 2 days, or 5 days later, mice were transcardially perfused with PBS. Brains were gently removed from the skull and embedded in O.C.T. (Sakura Finetek, AJ Alphen aan den Rijn, The Netherlands). For immunohistochemistry labeling, brains were cut into 10-μm-thick coronal cryosections and fixed in ice-cold acetone. Sections were stained overnight with antibodies against GFAP (Sigma; 1:800) and CD11b (ImmunoTools; 1:100). Then, the slices were incubated with the corresponding secondary antibodies at room temperature for 1 h (Cy2-conjugated donkey anti-mouse, Cy2-conjugated donkey anti-rabbit, and Cy3-conjugated donkey anti-rabbit, Jackson ImmunoResearch; 1:1000). Finally, the brain sections were analyzed under an inverted confocal microscope.

### Cell Culture

Cultures of primary mouse astrocytes were prepared from brains of postnatal (P0–P2) C57BL/6J mice as previously described [21]. Briefly, brain cortices were minced in cold PBS (pH 7.4) solution. The dissociation was completed by a 10-min incubation in 1 mM EDTA. The cells were plated and grown at 37 °C in DMEM supplemented with 10 % fetal bovine serum (FBS), penicillin (100 U/ml), and streptomycin (100 μg/ml) in a water-saturated atmosphere containing 5 % CO_2_. After 4 days, the medium was replaced by a fresh one, which was further replaced twice a week. After 10–14 days, the cultures reached confluence and the microglial cells were detached by incubating the plates twice on an orbital shaker during 8 h (200 rpm). Cells were then trypsinized, and CD11b-positive microglia were sorted out on a magnetic cell-sorting (MACS) device (Miltenyi Biotec, Bergisch Gladbach, Germany) as described previously [21, 22]. The flow-through consisted in negatively selected astrocytes, which were plated during 4 to 7 days in DMEM supplemented with 10 % FBS.

Primary cultures of neurospheres were obtained from murine embryonic neural stem cells derived from the ventricular zone (VZ) of embryonic day-14 (E14) C57BL/6J mouse embryos (Harlan) as described previously [23, 24].

Neurospheres were cultured in Neurobasal medium supplemented with 2 % B27 w/o vitamin A, 2 mM glutamine, penicillin (100 U/ml), streptomycin (100 μg/ml), and 20 ng/ml mouse recombinant epidermal growth factor (EGF). Neurospheres were differentiated into astrocytes by plating them on poly-l-ornithine-coated flasks filled with DMEM containing 10 % FBS. The cultures reached confluence within 2 weeks. The inflammatory stimulus was triggered by adding TNF (50 ng/ml) on the cells for 24 h. For semi-clonal experiments of neurosphere-forming assay, after TNF treatment, astrocytes were dissociated and plated in a 96-well plate (20 cells per well) in Neurobasal medium supplemented with B27 w/o vitamin A, 2 mM glutamine, penicillin (100 U/ml), streptomycin (100 μg/ml), and 20 ng/ml mouse recombinant EGF. After 1 month, neurospheres (bigger than 100 μm) were counted.

### NF-κB Inhibition

The NF-κB pathway was inhibited using 20 μM of 4-methyl-*N*^1^-(3-phenyl-propyl)-benzene-1,2-diamine (JSH-23) (Calbiochem, Merck, Darmstadt, Germany), a selective inhibitor of nuclear translocation of NF-κB p65. JSH-23 was added to cells 30 min prior to TNF treatment. The control condition used for JSH-23 was the vehicle (DMSO 0.05 %).

### Real-Time PCR Gene Expression Analysis

Total RNA was extracted by using the Invisorb Spin Cell RNA Mini Kit (Invitek, Berlin, Germany) or RNA NOW reagent (Biogentex, Seabrook, TX). For the in vivo experiments, biopsies of the injection area were collected (biopsy punch, 2 mm in diameter and 1 mm in depth, Miltex) and RNA extracted. The yield and integrity of RNA samples were assessed on a NanoDrop1000 device (Thermo Fisher Scientific, Waltham, MA, USA) and a microelectrophoresis device (Experion RNA StdSens Analysis Kit; Bio-Rad Laboratories, Hercules, CA, USA). The RNA was reverse transcribed into cDNA using Im-Prom-II Reverse Transcription System (Promega, Madison, WI, USA). Polymerase chain reactions (PCR) were set up in 20-μl reaction volume using PerfeCta SYBR Green SuperMix for iQ (Quanta Biosciences, Gaithersburg, MD, USA). Primers for each candidate gene were designed using Beacon Designer software (Premier Biosoft International, Palo Alto, CA, USA) and used at a final concentration of 500 nM. (See supporting information Table 1 for a list of primers used.) The relative changes of gene expression were estimated and normalized to β-actin by using the 2^−∆∆C^_T_ method.

**Table 1:** qPCR primers

	Forward	Reverse
Actb	5′-AGGGAAATCGTGCGTGACATCAAGAG-3′	5′-GGAGGAAGAGGATGCGGCAGTGG-3′
Oct4	5′-ACCACCATCTGTCGCTTC-3′	5′-CTCATTGTTGTCGGCTTCC-3′
Cd44	5′-TGGCACTGGCTCTGATTC-3′	5′-GTCTCTGATGGTTCCTTGTTC-3′
Gfap	5′-GGTTGAATCGCTGGAGGAG-3′	5′-CTGTGAGGTCTGGCTTGG-3′
Egfr	5′-TAATGTCTGCCACCTATGC-3′	5′-GCCACCACCACTATGAAG-3′
Sox2	5′-CGCAGACCTACATGAACG-3′	5′-TCGGACTTGACCACAGAG-3′
GlyPH	5′-GCTGCTCAACTGCCTACACATT-3′	5′-AACAGTCCTGGGCACAAAGG-3′
Ptg	5′-TCGCAGAGTGAGTGGAA GAGC-3′	5′-CTTGGAGTCCGCAAACACG-3′

### Immunolabeling

Cells were cultured on poly-l-ornithine-coated coverslips for 24 h and then fixed with 4 % paraformaldehyde for 20 min and permeabilized in PBS containing 0.3 % Triton X-100 (Sigma, St. Louis, MO, USA). The blocking was done using a PBS solution containing 3 % bovine serum albumin (BSA) (Sigma). The cells were then incubated overnight at 4 °C with polyclonal cyanine 3-conjugated mouse anti-GFAP IgG (1:800) (Sigma), rabbit anti-GFAP IgG (1:800) (Dako, Glostrup, Denmark), mouse anti-Nestin IgG (1:200) (Chemicon, Millipore, Billerica, MA, USA), and mouse anti-MAP2 IgG (1:200) (Chemicon). On the next day, the coverslips were incubated with species-specific secondary antibodies conjugated to cyanine 2 (Cy2-conjugated donkey anti-mouse, Jackson ImmunoResearch, West Grove, PA, USA) and cyanine 3 (Cy3-conjugated donkey anti-mouse or Cy3-conjugated donkey anti-rabbit, Jackson ImmunoResearch) (1:1000) for 1 h at room temperature. A biotin–streptavidin signal amplification strategy was performed for low expression as Oct4, CD44, Musashi-1, and EGFR. Then, the cells were stained with polyclonal rabbit anti-Oct4 IgG (1:100) (Abcam), rabbit anti-CD44 IgG (1:50) (Abcam), rabbit anti-Musashi-1 (1:100) (Abcam), and rabbit anti-EGFR (1:50) (Abcam) and sequentially incubated with biotinylated anti-rabbit secondary antibodies (1:1000) (Jackson ImmunoResearch) and Cy2-conjugated streptavidin. Preparations were counterstained with DAPI (1:10,000, Molecular Probes) and mounted with Aquamount on glass slides (Southern BioTech). Photomicrographs were acquired with a LSM 510 META inverted confocal microscope (Carl Zeiss Micro Imaging, Göttingen, Germany).

### Microarray Experiments and Quality Control

Cultures of primary mouse astrocytes were treated with TNF (50 ng/ml) for 24 h. Cells were collected and immediately homogenized in cooled-down RNA NOW reagent (OZYME). Total RNA was extracted according to RNA NOW manufacturer’s recommendations with −20 °C overnight incubation for small RNA precipitation. Total RNA integrity and purity were assessed using the Agilent 2100 Bioanalyzer and RNA 6000 Nano LabChip kits (Agilent Technologies). Only good-quality RNA (no contamination or degradation, RIN > 9) was used and further processed. Total RNA samples were reverse transcribed to double-stranded cDNA using specific primers, which reduce the priming of rRNA. cRNA was generated by in vitro transcription and reverse transcribed into a sense single-stranded cDNA. The cDNA was fragmented, labeled, and hybridized onto Affymetrix GeneChip Mouse Gene 1.0 ST arrays according to the Ambion Whole Transcript Expression kit for Affymetrix GeneChip Whole Transcript Expression Array Protocol (P/N 4425209 Rev.B 05/2009) and GeneChip WT Terminal Labeling and Hybridization User Manual for use with the Ambion Whole Transcript Expression kit (P/N 702808 Rev.6). Microarrays were then washed, stained, and scanned according to the manufacturer’s instructions. The six CEL files generated by the scanner were then imported into Partek Genomics Suite (GS) 6.4 for preprocessing and quality control. Preprocessing aims at estimating transcript cluster expression values from probe signal intensities. Thus, Partek options were set up for GC content adjustment, robust multiarray background correction, quantile normalization, log2 transformation, and mean summarization. Quality control was assessed through different methods available in Partek GS and did not reveal any outlier. Microarray expression data are available at the GEO database.

### Statistical Analysis and Data Visualization

Data are represented as mean ± standard error of the mean (SEM). Statistical differences between two groups were determined by nonparametric Mann–Whitney test. Multiple group comparisons were made using Kruskal–Wallis test with a significance level of 95 % followed by Dunn’s post test.

The real-time PCR expression data of time series were analyzed using the R software (R Foundation for Statistical Computing; Vienna, Austria). A two-way ANOVA, testing the effect of “treatment” and “time,” was performed on the delta Ct values (the residuals were tested to be normally distributed and delta Ct values of each group to be homoscedastic). If a factor of more than two levels significantly affected the dCt values, a Tukey post hoc test was used to determine the source of the variation. Similarly, the in vivo data were analyzed by a two-way ANOVA testing the effect of the factors “treatment” and “hemisphere.”

Microarray gene expression data was normalized using the GC-RMA procedure with default parameters for background correction, quantile normalization, and probe replicate summarization [25]. Differentially expressed genes between control and TNF conditions were then determined using the empirical Bayes moderated *t* statistic (eBayes) [26]. *p* value significance scores for these genes were adjusted for multiple hypothesis testing according to the Benjamini–Hochberg procedure [27].

A heat map and dendrogram cluster visualization for the top 100 most significant known genes (see Fig. 2a) was obtained using standard hierarchical average linkage clustering with a Euclidean distance metric. To create network visualizations for the two marker genes of interest, GFAP and glycogen phosphorylase (Pygb), interactions of the corresponding proteins were retrieved from the STRING database [28] using only protein–protein interactions with a minimum confidence score of 900 out of a maximum of 1000 (see Fig. 2b, c). Next, the layout of the network representations was generated by applying 1000 iterations of the Fruchterman–Reingold automated graph layout algorithm [29]. Finally, nodes in these network graphs were colored such that underexpressed genes in the TNF samples as compared to the control samples are represented by blue nodes and overexpressed genes by red nodes (the color darkness is proportional to the absolute fold change on a logarithmic scale).

Alterations in known cellular pathways and processes were identified and visualized by applying the MetaCore™ GeneGO software onto the differential expression statistics obtained from the eBayes analysis (see above). The genes were pre-filtered using a significance threshold (adjusted *p* value <0.05) before applying the default GeneGO pathway analysis. In the resulting pathway visualizations, underexpressed genes in the treated samples are highlighted by a blue bar and overexpressed genes by a red bar (the length of the colored bar next to each gene represents the absolute fold change on a logarithmic scale).

## Results

### TNF Induces the Re-expression of Markers of Stemness State in Neurosphere-Derived Astrocytes

**Fig. 1:**
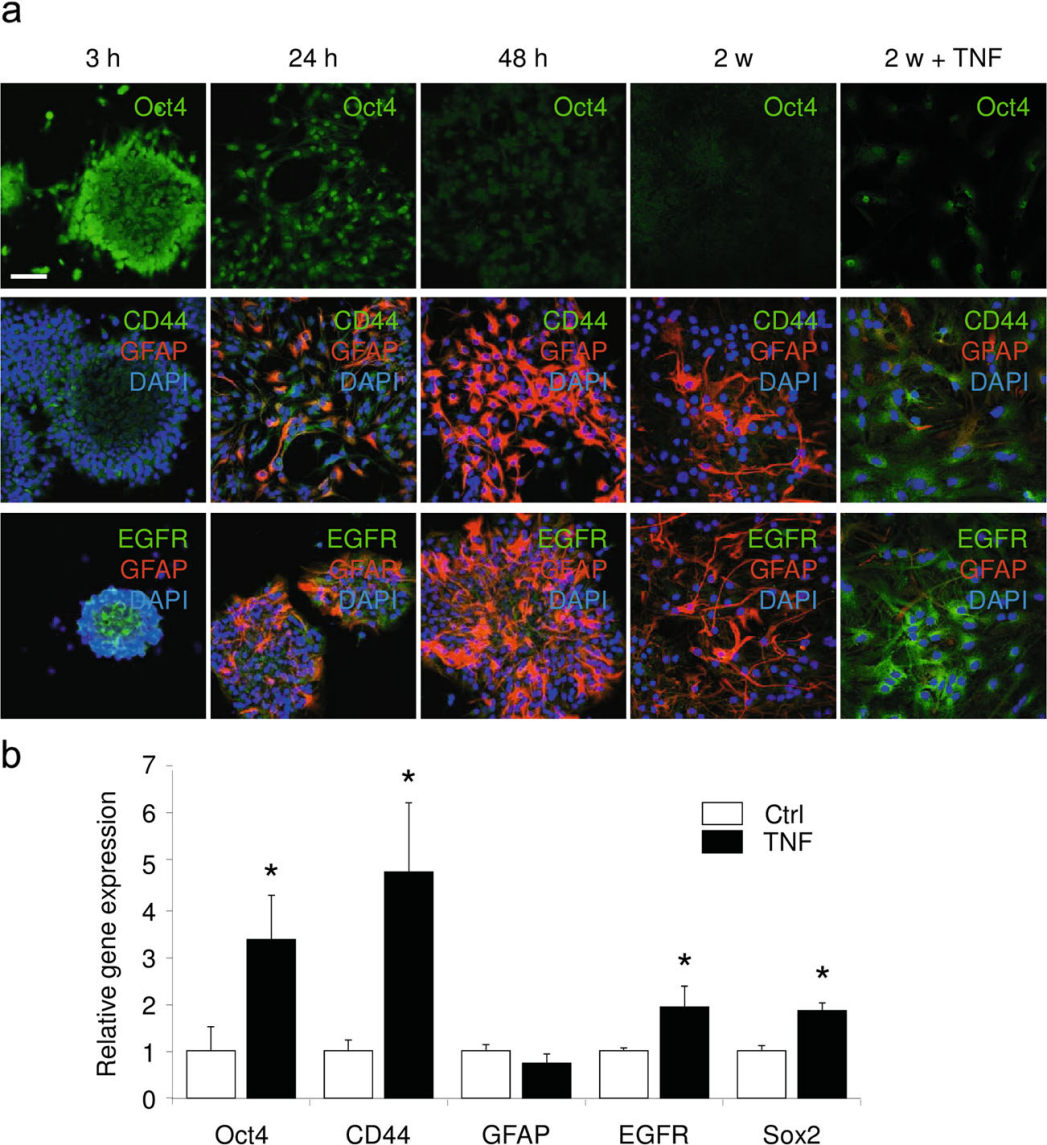
**a** NSC differentiation to astrocytes. After 3 h, 24 h, 48 h, 2 weeks, and 2 weeks + TNF (last 24 h TNF treatment, *last column*) of neurosphere differentiation, cells were analyzed by immunofluorescence for the expression of Oct4 (*upper panel*, *green*), GFAP (*middle panel*, *red*), and CD44 (*middle panel*, *green*). In the *lower panel*, cells were stained for GFAP (*red*) and EGFR (*green*). *Scale bar* = 50 μm. **b** TNF induces the re-expression of markers of stemness state in neurosphere-derived astrocytes. Real-time PCR was used to assess the regulation of Oct4, CD44, GFAP, EGFR, and Sox2 in neurosphere-derived astrocytes. Gene expression has been normalized to β-actin. *n* = 3; *error bars* represent the SEM. *p < 0.05

In order to determine whether inflammation could convert astrocytes to a more immature state, we first studied the differentiation kinetics of neural progenitors to astrocytes by protein expression levels of stemness markers. As shown in previous studies, neurospheres differentiated in the presence of 10 % FBS finally form a flat layer of astrocytes [23]. During the first days of differentiation, the expression of stemness markers, such as Oct4, CD44, and EGFR, decreased and was correlated to an increase in GFAP expression (Fig. 1a). Oct4, CD44, and EGFR were detected on progenitor cells in vitro. After 3 h of differentiation, all progenitor cells (in the periphery of the neurospheres) were still positive for Oct4, CD44, and EGFR staining (Fig. 1a). After 1 day of differentiation, even though the cells began to express GFAP, we could observe that the expression of Oct4 and CD44 persisted in astrocyte-restricted precursor cells (Fig. 1a). In contrast, after 2 days of differentiation, the majority of differentiated cells expressed GFAP, while losing the expression of stemness markers. At this time point, Oct4, CD44, and EGFR were no longer detected (Fig. 1a). These results show that stemness markers such as Oct4, CD44, and EGFR expressed in neural stem cells and progenitor cells persist in astrocyte-restricted precursor cells but are absent in GFAP-expressing astrocytes.

In order to determine the effect of inflammation on differentiated astrocytes, neurosphere-derived astrocytes were treated with TNF after 2 weeks of differentiation. In untreated and TNF-treated neurosphere-derived astrocytes, Oct4, Sox2, CD44, EGFR, and GFAP transcript expression levels were measured by real-time PCR. We observed important modulations in Oct4, Sox2, CD44, and EGFR mRNA expression when cells were activated with TNF (Fig. 1b). After 24 h, Oct4, Sox2, CD44, and EGFR expression increased in TNF-activated cells (Fig. 1b). In contrast, GFAP expression decreased slightly under the same conditions (Fig. 1b). At the protein level, we were also able to observe the re-expression of stemness markers following TNF treatment (Fig. 1a). These results show that TNF induces the conversion of mature astrocytes into a more immature state.

### TNF Induces the Re-expression of Markers of Stemness in Primary Astrocytes

**Fig. 2:**
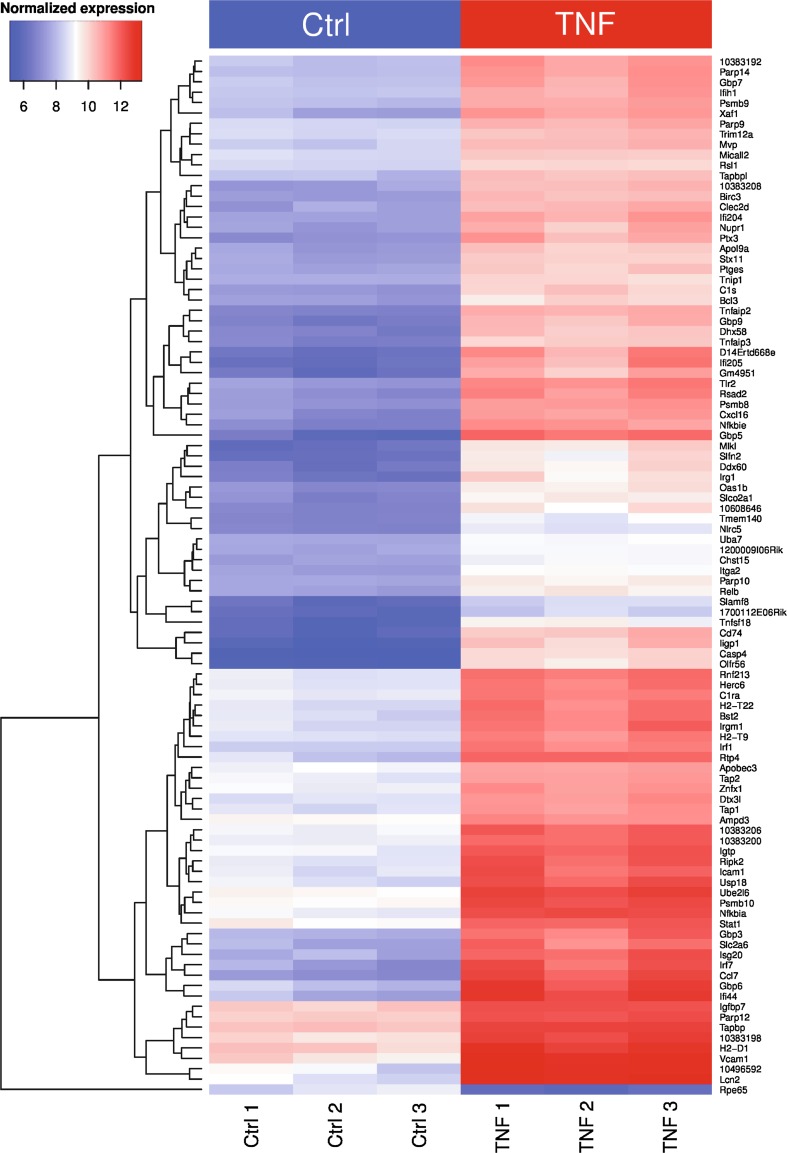
Heat map visualization of the normalized gene expression levels for the top 100 most significant known genes with differential expression between control and TNF samples according to the empirical Bayes moderated *t* statistic [26]. Hierarchical clustering was applied to identify groups of genes with similar expression profiles (see dendrogram visualization on the *left*)

**Fig. 3:**
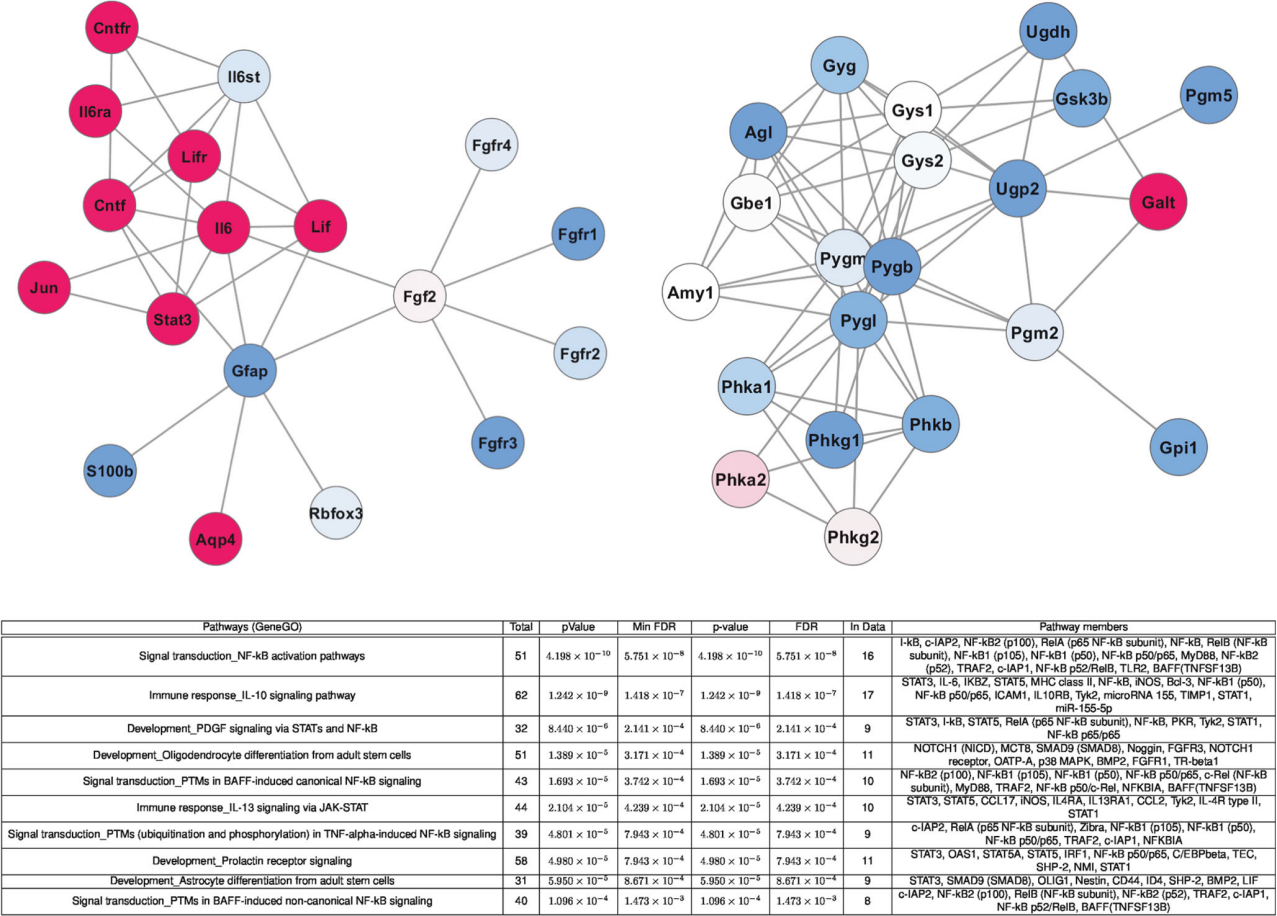
For the two marker genes of interest, GFAP and glycogen phosphorylase (Pygb), the local interaction network for the corresponding proteins was determined using protein–protein interactions from the STRING database [28] (with a confidence score of at least 900). Graph representations of these interaction networks were created using 1000 iterations of the Fruchterman–Reingold automated graph layout algorithm [29]. *Blue nodes* represent underexpressed genes in the TNF samples as compared to the control samples, and *red nodes* highlight overexpressed genes (*darker colors* reflect larger absolute fold changes on a logarithmic scale). Cellular pathways and processes identified using the GeneGO pathway analysis of known genes with significant differential expression between control and TNF samples

**Fig. 4:**
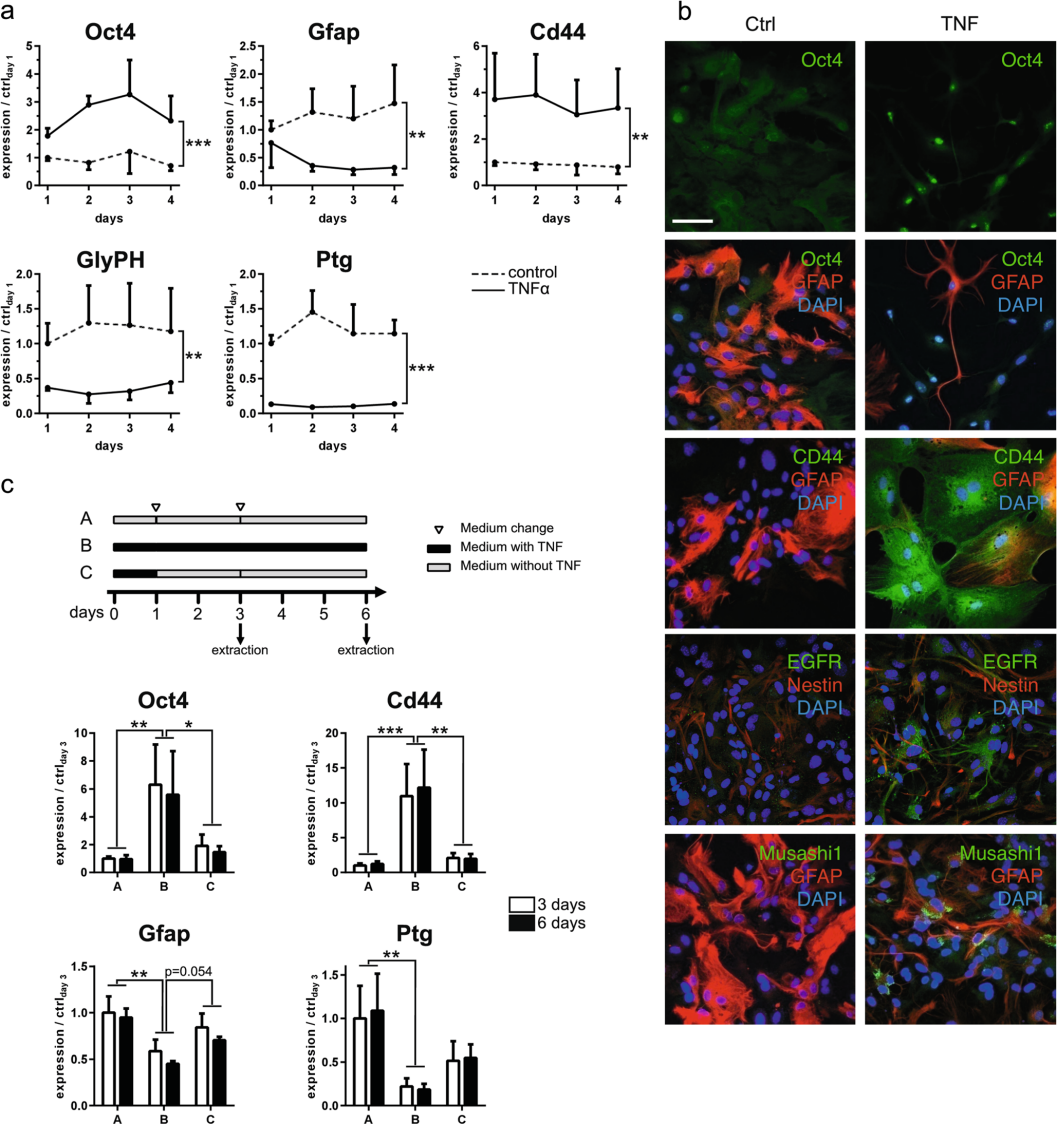
TNF induces astrocyte dedifferentiation. **a** RNA of primary astrocytes were collected 24 h, 48 h, 3 days, and 4 days after TNF treatment and analyzed for the expression of Oct4, CD44, GFAP, glycogen phosphorylase, and PTG by real-time PCR. Gene expression has been normalized to β-actin. *n* = 3; *error bars* represent the SEM. **b** Primary astrocytes were treated with TNF for 24 h and then fixed and stained for immunofluorescence. From the *upper to the lower panels*, we analyzed the expression of Oct4 and CD44 (*green*) compared to GFAP (*red*). Primary astrocytes were treated with TNF for 3 days and then fixed and stained for immunofluorescence. We analyzed the expression of Musashi-1 and EGFR (*green*) compared to GFAP and Nestin (*red*). *Scale bar* = 50 μm. **c** RNA of primary astrocytes were collected 3 and 6 days after the initial 24-h TNF treatment and cultivated with or without TNF up to RNA extraction and analyzed for the expression of Oct4, CD44, GFAP, and PTG by real-time PCR. Gene expression has been normalized to β-actin. *n* = 3 independent experiments; *error bars* represent the SEM. *p < 0.05, **p < 0.01, ***p < 0.001

Although neurosphere-derived astrocytes can be considered as a microglia-free alternative to primary astrocyte cultures, we wanted to confirm our observations on mixed glial culture derived-astrocytes. Mixed glial cultures contain mostly astrocytes but also a variable amount of contaminating microglia (CD11b-positive cells). We negatively selected astrocytes with the MACS technology by using anti-CD11b antibody-coupled microbeads to eliminate the microglial cell population. To confirm the previous results obtained with neurosphere-derived astrocytes, we treated primary astrocytes with TNF (50 ng/ml for 24 h). Microarray analysis of primary astrocytes treated for 24 h with TNF showed that the NF-κB pathway and its related inflammatory signaling were strongly induced (Fig. 2). Moreover, pathway analysis with GeneGO revealed that pathways related to glial differentiation were modulated (Fig. 3). Network visualizations of two marker genes of interest, GFAP and glycogen phosphorylase (Pygb), highlighting interactions of the corresponding proteins and expression alterations in their coding genes, confirmed that the astrocyte phenotype is changing under inflammatory conditions (Fig. 3). Using real-time PCR, we checked whether TNF initiates the conversion of mature astrocytes to a more immature state. As for neurosphere-derived astrocytes, we could observe that on primary astrocytes, a TNF treatment was able to significantly upregulate Oct4 and CD44 expression, as well as downregulate GFAP, *glycogen phosphorylase*, and PTG expression (Fig. 4a). Moreover, this change in expression of Oct4, CD44, GFAP, *glycogen phosphorylase*, and PTG, induced by TNF, was maintained during 4 days (Fig. 4a). The analysis of TNF-treated primary astrocytes by confocal microscopy revealed that the activation of the NF-κB pathway for 24 h was sufficient to induce the expression of typical neural progenitor cell (NPC) markers such as Oct4, EGFR, Musashi-1, and CD44 while GFAP expression diminished (Fig. 4b). On the other hand, untreated astrocytes did not express these NPC markers with a large number of cells remaining GFAP positive (Fig. 4b). Interestingly, we were not able to observe any change in Nestin expression after 24 h of TNF activation (Fig. 4b). When TNF was removed after 24 h of treatment, expression levels of Oct4, CD44, GFAP, and PTG after 2 and 5 days returned towards normal levels (Fig. 4c). To estimate the fraction of cells that are able to proliferate after 24 h of TNF treatment, we counted Ki67-positive cells. No difference was observed between control and TNF-treated astrocytes (4.7 % in control conditions and 2.9 % in TNF-treated astrocytes).

**Fig. 5:**
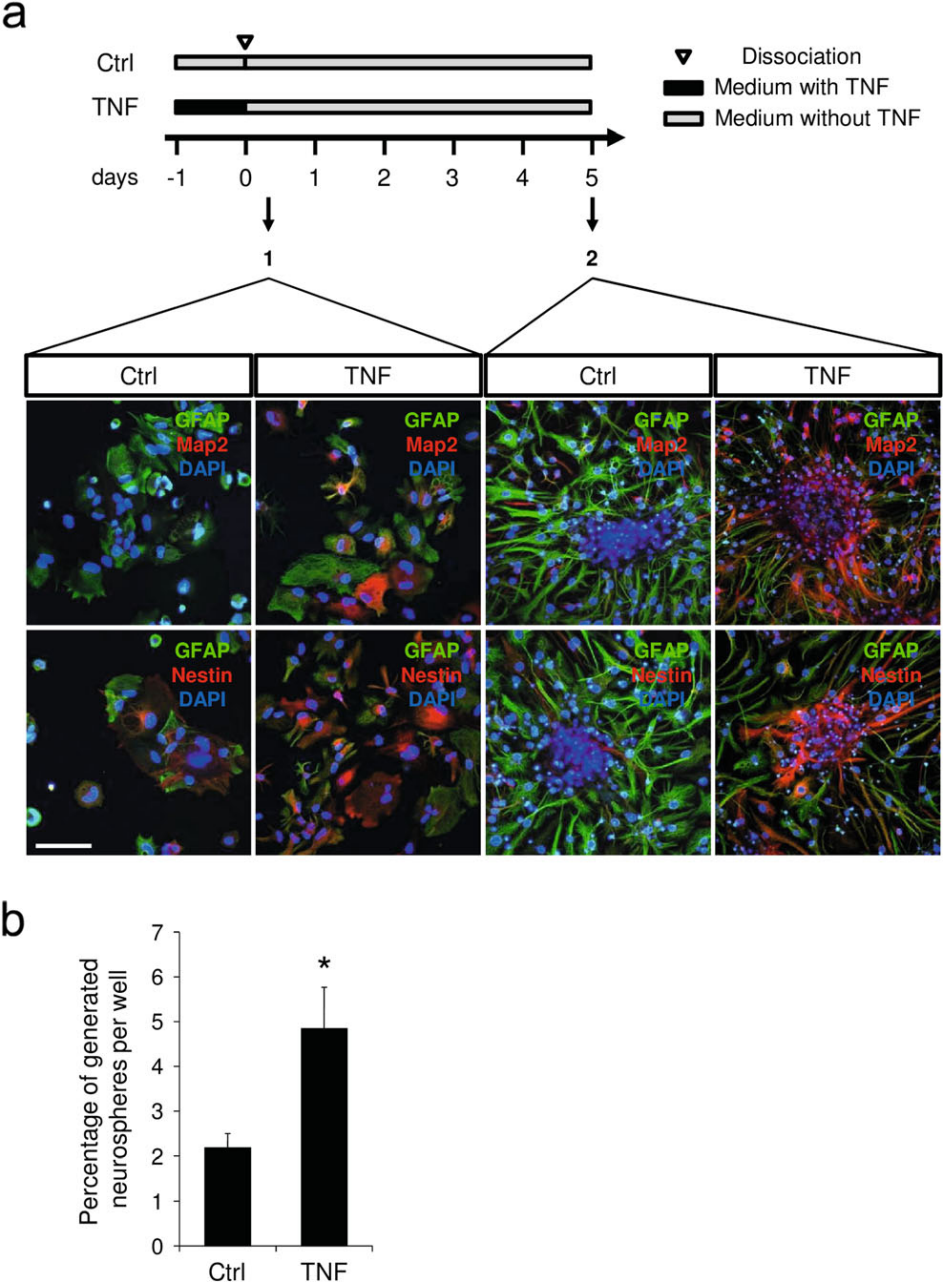
TNF treatment results in the appearance of cells with neural progenitor phenotype. **a** Primary astrocytes were treated with TNF for 24 h, dissociated, and re-plated in Neurobasal medium containing EGF (experimental setup). Afterwards, cells were analyzed by immunocytochemistry. The expression of MAP2 (2A + 2B) and Nestin (both in *red*) were compared to that of GFAP (*green*) after 6 h (*1*) or 5 days (*2*). *Scale bar* = 50 μm. **b** Semi-clonal experiments of the neurosphere-forming assay. After TNF treatment, astrocytes were dissociated and plated in a 96-well plate (20 cells per well) in neurosphere medium. After 1 month, neurospheres were counted. *n* = 3 independent experiments; *error bars* represent the SEM. *p < 0.05

To demonstrate that astrocytes treated with TNF present some characteristics of neural progenitors, we examined the differentiation potential of cells reacting to inflammation using a neurosphere-forming assay (see Fig. 5a for experimental design). Whereas few neurospheres were generated from cells isolated from a primary astrocyte culture, cells isolated from TNF-treated cultures were able to form higher amounts of neurospheres. In fact, quantitative data obtained from semi-clonal neurosphere-forming assays show that the percentage of cells able to generate a neurosphere is doubled in astrocytes treated with TNF in comparison to control (Fig. 5b). After dissociation of primary astrocytes, cells treated for 24 h with TNF and then cultivated for 6 h in neurosphere medium (containing EGF) expressed MAP2 (2A + 2B) and Nestin (Fig. 5a). After 5 days in neurosphere medium, many MAP2 (2A + 2B) and Nestin-positive cells were observed in cultures previously treated with TNF (26.2 % of cells were MAP2 positive and 32.9 % of cells were Nestin positive) (Fig. 5a). In control conditions, few cells expressed MAP2 (2A + 2B) and Nestin (5 % of MAP2-positive cells and 8.6 % of Nestin-positive cells) (Fig. 5a). These observations confirm that in primary cultures of astrocytes activated with TNF, some cells are able to respond to neurosphere medium and produce neural progenitors (Nestin-positive cells) and neurons (MAP2-positive cells). The absence of oligodendrocyte progenitor cells (identified with O4 antibody) during the neurosphere assay suggests that neural progenitor cells obtained after dedifferentiation of astrocytes are bipotent. Similar results were obtained with neurosphere-derived astrocytes, which represent a model of pure astrocyte culture. Taken together, our in vitro observations show for the first time that after TNF treatment, a subset of astrocytes dedifferentiates towards a more immature state characterized by the re-expression of stemness markers and the repression of typical astrocyte markers such as GFAP, glycogen phosphorylase, and PTG. This dedifferentiation process is maintained as long as TNF is present in the culture medium. Once TNF is removed, astrocytes recover their initial phenotype and are undistinguishable from control astrocytes.

### Inflammation Induces Dedifferentiation of Astrocytes In Vivo

**Fig. 6:**
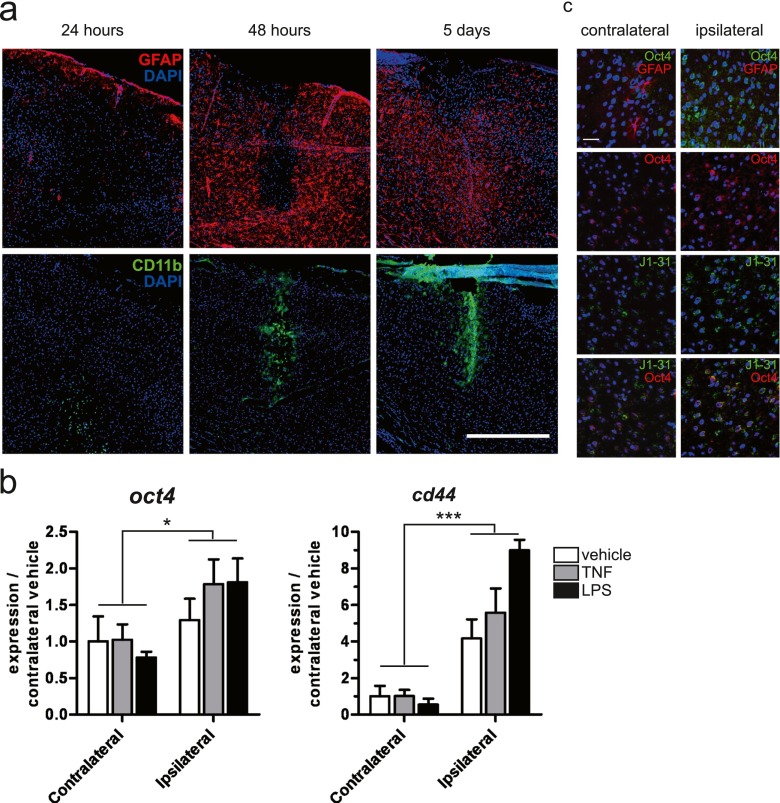
Inflammation induces astrocyte dedifferentiation in vivo. **a** Stab wound injury was executed with PBS in the right primary somatosensory cortex using a Hamilton syringe (1 μl). 24 h, 48 h, and 5 days after stab wound with PBS using a Hamilton syringe (1 μl), brains were collected, sliced, and analyzed by immunofluorescence for the expression of GFAP (*red*) and CD11b (*green*). *Scale bar* = 500 μm. DAPI nucleus staining in *blue*. **b** Stab wound injury was executed with PBS or LPS (1 μg/μl) or TNF (10 ng/μl) in the right primary somatosensory cortex using a Hamilton syringe (1 μl). Tissue samples were collected using a biopsy patch and followed by RNA extraction, 24 h after wounding. Samples were analyzed for the expression of Oct4 and CD44 by real-time PCR. Gene expression has been normalized to β-actin. *n* = 3 animals per experimental group; *error bars* represent the SEM. *p < 0.05 and ***p < 0.001 for the main effect “hemisphere” in the two-way ANOVA (hemisphere × treatment). **c** 24 h after stab wound with PBS using a Hamilton syringe (1 μl), brains were collected, sliced, and analyzed by immunofluorescence for the expression of Oct4 (*upper panel*, *green*; *middle and lower panels*, *red*), GFAP (*upper panel*, *red*), and J1-31 (anti-astrocyte antibody) (*middle and lower panels*, *green*). Ipsilateral sides are compared to contralateral sides. *Scale bar* = 50 μm. DAPI nucleus staining in *blue*. *p < 0.05, ***p < 0.001

To confirm that the activation of the inflammatory signaling pathway directs the dedifferentiation of astrocytes in vivo, we realized a kinetic experiment of cortical stab wound lesions in adult mice. Animals were sacrificed 24 h, 48 h, and 5 days after injury. Frontal sections were realized in order to assess the phenotype of cells present in the area of the lesion. After 24 h, on low-magnification images, CD11b-positive cells corresponding to microglia were already present within the site of lesion while no GFAP-positive cells were observed. Nevertheless, some GFAP-positive cells could be observed at distance of this area (Fig. 6a). It is noteworthy that the basal GFAP staining is weak in the healthy mouse cortex. The absence of GFAP-positive cells in close proximity to the stab wound lesion persisted during 48 h while the expression of GFAP increased around this site (Fig. 6a). Five days after injury, the glial scar seemed completely present as many reactive astrocytes expressed large amounts of GFAP (Fig. 6a). Concerning transcript analysis, animals were sacrificed 24 h after the injury to collect biopsies of the lesioned area and to extract RNAs. Oct4 and CD44 expression was examined by real-time PCR. Transcript levels of Oct4 and CD44 were upregulated in the area of the lesion (ipsilateral) compared to the contralateral area (control) (Fig. 6b). Frontal sections were also realized 24 h after the injury in order to assess the phenotype of cells present in the area of the lesion. Consistent with our in vitro data, we were able to observe Oct4-positive cells in the lesioned area (Fig. 6c) in parallel to a decrease of GFAP-positive cells (Fig. 6c). The use of an astrocyte/low-grade astrocytoma marker demonstrated that Oct4 is re-expressed in astrocytes during injury. In fact, this monoclonal antibody (Mab JI-31) recognizes an intracellular protein antigen expressed by astrocytes and upregulated in reactive astrocytes [30]. Consistent with our hypothesis, the Oct4-positive cells were costained with the J1-31 anti-astrocyte antibody in the area of the lesion (Fig. 6c). Overall, our in vivo data suggest that in the area of injury during the very early response, inflammation induces a transient decrease of GFAP expression in astrocytes correlated with the re-expression of stemness markers. These observations confirm our previous in vitro results.

### NF-κB inhibition Antagonizes the TNF-Induced Dedifferentiation of Reactive Astrocytes In Vitro

**Fig. 7:**
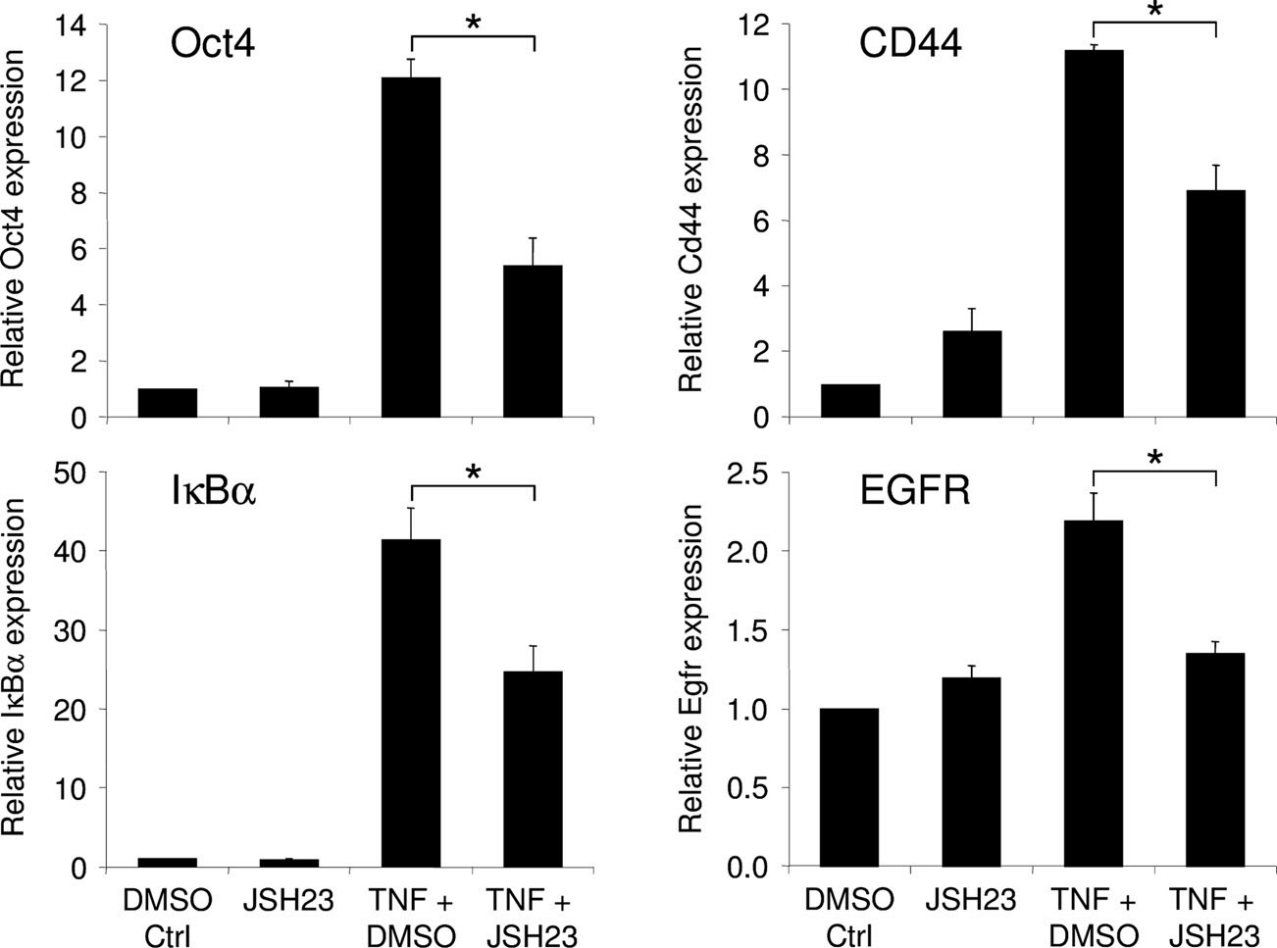
Effects of NF-κB inhibition on the dedifferentiation of astrocytes. The NF-κB inhibitor JSH-23 was added 30 min prior to 24 h of TNF treatment on primary astrocytes. Real-time PCR was used to assess the regulation of Oct4, CD44, IκBα, and EGFR. Gene expression has been normalized to β-actin. *n* = 3; *error bars* represent the SEM. *p < 0.05

Previous results have shown that the aromatic diamine JSH-23 compound has an inhibitory effect on the NF-κB transcriptional activity [31]. The NF-κB inhibitory activity of the JSH-23 compound in our cells was shown by the reduced expression of IκBα which reflects the activity of the NF-κB pathway (Fig. 7). To demonstrate that the dedifferentiation induced by TNF (50 ng/ml) in astrocytes was dependent on NF-κB activity, we used JSH-23 (20 μM). After 24 h of TNF activation, primary astrocytes treated with JSH-23 presented a low re-expression of *Oct4*, *CD44*, and *EGFR* when compared to cells treated with TNF alone (Fig. 7). Our data demonstrate that the dedifferentiation of astrocytes by TNF depends at least partly on the NF-κB pathway.

## Discussion

Beside the well-established sources of neural stem cells (NSCs), it was recently shown that astrocytes could be a source of multipotent cells in the brain [9, 10]. After injury, during reactive gliosis, some astrocytes acquire stem cell properties. Reactive gliosis is the reaction to brain injury, but the precise origin of the glial cells implicated in this response is still unknown. Astrocytes react to injury by undergoing a phenotypical transformation characterized by hypertrophy and cytoskeletal modifications like the upregulation of GFAP and the re-expression of the progenitor markers vimentin and Nestin. While it is generally accepted that mature astrocytes do not normally divide, a subpopulation of reactive GFAP-positive cells does so, prompting the question of whether the proliferating GFAP-positive cells arise from endogenous glial progenitors or from mature astrocytes that start to proliferate in response to brain injury. Yet, reprogramming or potential differentiation was already previously suggested [8] and the work of Buffo and co-workers shows that mature astrocytes start to proliferate after a stab wound injury and contribute to the reactive gliosis and proliferation of GFAP-positive cells. This conversion of somatic cells, like astrocytes, to a multipotent state by reprogramming, raises a number of mechanistic questions. Sharif et al. recently demonstrated that TGFα promotes the sequential conversion of mature astrocytes into neural progenitors [11]. In addition, Yang et al. showed that astrocyte dedifferentiation is induced in a scratch wound assay or by conditioned culture medium collected from scratch assay astrocytes [12].

In the present study, we provide evidence that TNF-induced NF-κB stimulation results in the dedifferentiation of astrocytes in vitro. GFAP is the classical marker used to identify differentiated astrocytes [32]. However, functional features of mature astrocytes may represent better markers of terminal differentiation for astrocytes. Glycogen is the major energy reserve of the brain and it is exclusively localized in astrocytes [33]. After 24 h of TNF stimulation in vitro, the phenotype of mature astrocytes has changed. In fact, astrocytes lose the expression of GFAP, glycogen phosphorylase, and PTG. For two marker genes of interest, GFAP and glycogen phosphorylase (Pygb), the local interaction network for the corresponding proteins confirms this action of TNF. Interestingly, the increase of expression of Jak-Stat signaling components (observed in the local interaction network for GFAP) after TNF stimulation could explain the loss of GFAP. In fact, overexpression of Jak-Stat signaling is known to inhibit astrogliogenesis and GFAP expression [34]. At the same time, a population of astrocytes re-express immaturity state markers, such as CD44, Musashi-1, and Oct4. In our conditions, neurosphere-derived astrocytes treated with TNF increase their expression of Oct4, CD44, EGFr, and Sox2 after 24 h. It is interesting to note that this dedifferentiation process is maintained as long as TNF is present in the culture medium. Once TNF is removed, astrocytes recover the phenotype of control astrocytes. When we incubated TNF-treated primary astrocytes in neurosphere growth medium containing EGF, we were able to obtain neural progenitors as well as neuron-like cells. This neurosphere assay enforces the hypothesis that TNF induces the conversion of some astrocytes into neural progenitors. Strikingly, the absence of oligodendrocyte progenitor cells in this assay suggests that the hereby obtained neural progenitors are bipotent and, once differentiated, are only able to generate neurons and/or astrocytes. Interestingly, Ravin et al previously described the existence of a bipotent neuron–astrocyte progenitor, which derives from the multipotent CNS stem cell [35]. In addition, the team of D. A. Steindler found similar bipotent progenitors, which were not able to give rise to oligodendrocytes, when placing human cells from the temporal lobe epilepsy area in growth conditions favoring the propagation of progenitors [36].

The same results are obtained with pure cultures of neurosphere-derived astrocytes. The use of two different models of astrocyte cultures supports the hypothesis of a dedifferentiation of astrocytes while making the possibility of a contamination with certain neural stem cell populations highly unlikely. In fact, we never could obtain neurospheres after dissociation and cultivation in neurosphere medium of neurosphere-derived astrocytes. These in vitro observations are, at least partially, confirmed in a mouse model of cortical lesions. While it is admitted that gliosis is accompanied by an increase of GFAP, detailed kinetic studies performed in our lab have shown that this increase of GFAP expression begins only 2 days after injury (Fig. 6a). Interestingly, we observed a transient decrease of GFAP after 24 h, which persists 48 h after the injury within the site of lesion. Together, our in vitro and in vivo data suggest that during the first step of reactive gliosis, there is a conversion of mature astrocytes into an immature state (GFAP negative, CD44 positive, and EGFR positive). It is interesting to note that CD44 identifies an astrocyte-restricted precursor cell that is committed to generate astrocytes in vitro and in vivo in both rodent and human tissues [37]. In this population of dedifferentiated astrocytes, some cells present the characteristic markers of neural progenitors (Oct4, Musashi-1) and are able to generate new neurons and astrocytes in a medium containing EGF. The use of the NF-κB inhibitor JSH-23 confirms that the observed dedifferentiation in the presence of TNF is clearly due to the activation of the classical NF-κB pathway. Moreover, the use of highly pure astrocyte cultures, derived from neurospheres, is arguing that astrocytes themselves respond to TNF and that we do not observe an indirect effect through a microglial cell release.

The conversion of somatic cells, like astrocytes, to a multipotent state raises a central question: which regulator triggers this process of dedifferentiation? Pluripotency is a transient feature that becomes increasingly restricted during development [38]. ESC self-renewal and differentiation are tightly regulated by specialized transcription factors such as Oct4, Sox2, and Nanog [18, 39]. Suppression of Oct4, Sox2, and Nanog has been correlated with cell fate specification and lineage-specific differentiation [40]. While Oct4 is highly expressed in ESCs, it becomes silent upon differentiation. Some studies show that Oct4 could be detected at a low level in various progenitor cells and in somatic tissues [41]. Interestingly, it has been shown that Oct4 is expressed in NSCs and neural progenitor cells and that tuning its expression influences the cell fate. Thus, downregulating Oct4 accelerates the neuronal differentiation of progenitor cells during development, while its sustained expression prevents neuronal differentiation [19]. Additionally, recent data demonstrate the differential expression of Oct4 in neural stem cells grown in vitro [42]. The authors found that the expression of Oct4 in the CNS is still active by E8.5 but declines rapidly until becoming undetectable by E15.5. This decline coincides with the gradual methylation of the Oct4 promoter and proximal enhancer [18]. These results indicate that Oct4 is an essential regulator of NSC differentiation during development. Recently, molecular beacons towards SOX2 and Oct4 mRNA were successfully used to track gene expression in living neurosphere cells [43]. In addition, recent studies show that Oct4 is highly expressed in human gliomas as well as in glioma cell lines, and that its expression level is positively correlated to increasing glioma grades [15, 20]. In cell cultures, it has been shown that Oct4 is expressed in rat C6 glioma cells and rat neural stem cells but not in differentiated cells from the rat brain. Strikingly, overexpression of Oct4 in C6 cells was able to increase the expression of Nestin and Stat3 phosphorylation while decreasing the expression of GFAP, which suggests that Oct4 might inhibit the differentiation of glioma cells [20]. Amazingly, recently, the presence of a rare population of Oct4-positive primitive neural stem cells is observed in the periventricular region of the adult mice forebrain whose progeny includes GFAP-positive type b cells [44]. Taken together, these results demonstrate that Oct4 could play a role not only in ESCs but also in more restricted progenitors in the CNS.

Interestingly, we observed that the conversion of mature astrocytes into progenitor-like cells correlated with the upregulation of Oct4 transcript levels, suggesting that Oct4 might be able to modulate the astrocyte phenotype in response to TNF. In addition, the nuclear localization of Oct4 in reactive astrocytes suggests that it might be active. The re-expression of Oct4 could be able to modulate the expression profile of astrocytes during TNF treatment. We tried to investigate this hypothesis and used siRNA to silence Oct4. Unfortunately, we failed to detect a significant change in these modulation trends. We nevertheless provide these results in the supplementary figure (SD4). Our observations sustain the central role of Oct4 as described in glioma cells [20] and suggest that the cellular mechanism occurring during the retinal regeneration in zebrafish (re-induction of pluripotency factors like Oct4 or Sox2 in Müller glia) could be partially conserved in mammalian CNS [45]. Epigenetic modifications might explain the reactivation of Oct4. In fact, recent data show that the activation of inflammatory pathways causes rapid and global changes in the expression of epigenetic modifiers, which enhance chromatin remodeling and nuclear reprogramming [46]. For example, the microRNA-145, a miRNA enriched in rat spinal neurons and astrocytes, is downregulated after spinal cord injury [47]. It is known that expression of microRNA-145 is low in self-renewing human embryonic stem cells (hESCs) but highly upregulated during differentiation and that the OCT4, SOX2, and KLF4 are direct targets of miR-145 [48]. Then, we could hypothesize that the downregulation of microRNA-145 might explain the reactivation of Oct4 in inflammatory conditions.

**Fig. 8:**
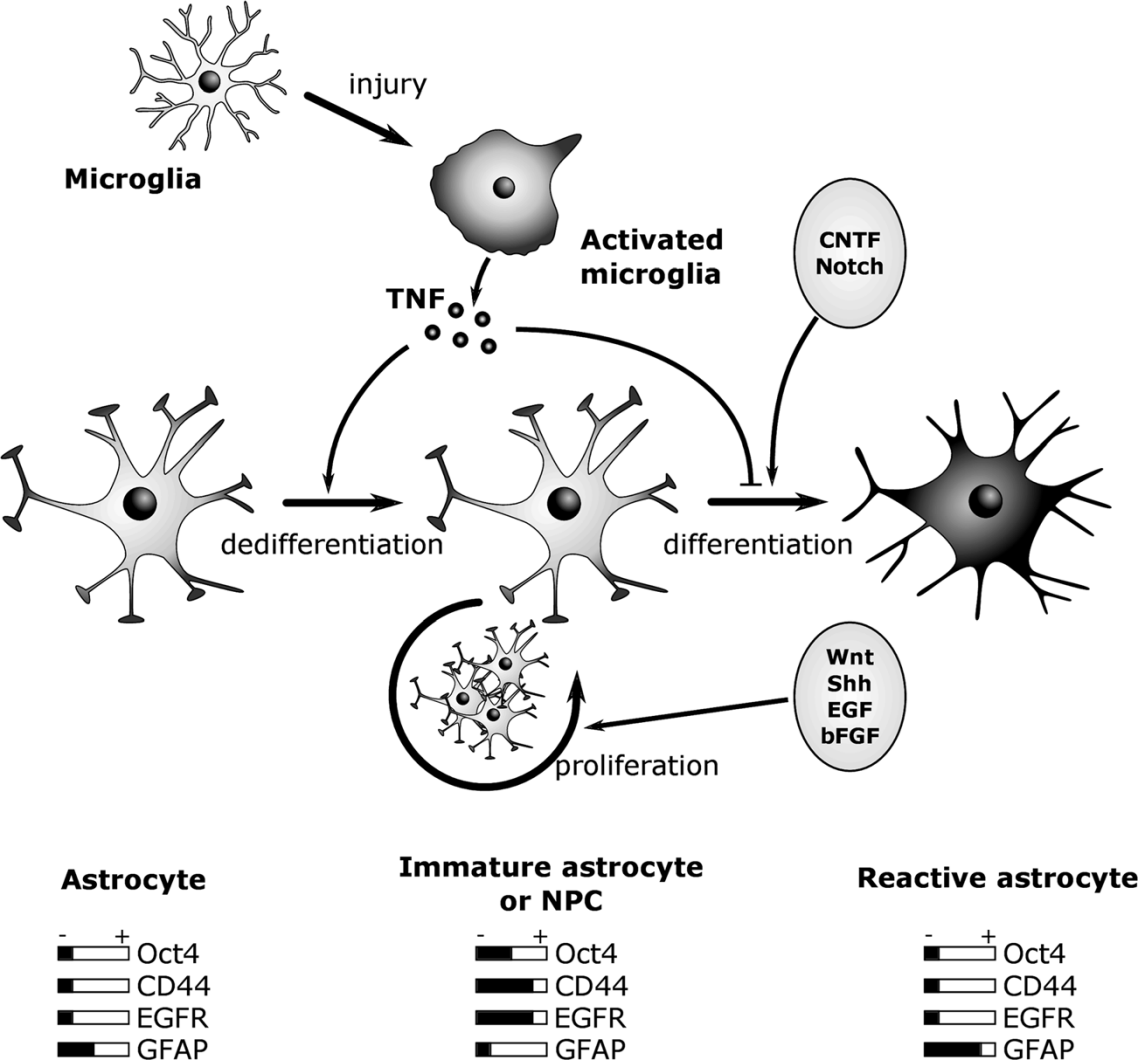
Proposed model for the role of TNF in the generation of reactive glia during injury. An initial microglia activation during injury gives rise to TNF production, which induces astrocyte dedifferentiation. TNF release maintains the dedifferentiated astrocyte in an immature state within the injury area. In this immature state, dedifferentiated astrocytes are able to proliferate after the stimulation of pathways like Wnt, SHH, EGF, or bFGF. When TNF decreases, reactive astrocytes could re-differentiate and express their typical marker, GFAP

This study uncovers the ability of astrocytes to undergo a functional dedifferentiation in response to an inflammatory stimulus. In fact, during an early response of reactive gliosis, TNF can induce the dedifferentiation of astrocytes by the re-expression of immaturity genes and the loss of marker of mature astrocytes like GFAP or glycogen metabolism gene expression. These dedifferentiated astrocytes are at least in part behaving like neural progenitors. Thus, they are able to proliferate in a medium containing EGF and can differentiate into both neurons and astrocytes. The molecular mechanisms underlying the dedifferentiation of astrocytes involve the NF-κB pathway as well as the transcription factor Oct4. Our model predicts that the re-expression of Oct4 modulates the expression of CD44 and GFAP and engages the conversion of a mature astrocyte to an immature state, which shares some characteristics with neural progenitors. The data presented here reveal the plasticity of astrocytes and a new role for Oct4 in the control of the dedifferentiation process of mature cells. Combining our results of the role of TNF in the dedifferentiation of astrocytes with the results already available in the literature enables us to propose a model of in vivo reactive gliosis, which we describe in Fig. 8. Initial microglia activation during injury gives rise to the production of TNF, which in turn induces the dedifferentiation of astrocytes. As long as TNF is released, these dedifferentiated astrocytes remain in an immature state in the vicinity of the injury. These dedifferentiated astrocytes might be able to proliferate under the stimulation of pathways like Wnt/β-catenin, SHH, EGF, and bFGF [10, 49, 50]. Once TNF decreases, reactive astrocytes could differentiate again in response to IL-6-type cytokines or the activation of the Notch signaling, and ultimately express their typical marker, GFAP [51, 52]. It would be interesting to investigate if the same dedifferentiation of astrocytes occurs in the facial nerve transection model, as it has been described that the response of astrocytes occurs at distance in a less inflammatory environment [53].

This study uncovers a remarkable capability of astrocytes to undergo a dedifferentiation process in response to a single change in their extracellular environment. Finally, our results suggest that the astrocytic dedifferentiation process observed during inflammation could have important implications on therapeutic strategies in injured CNS regeneration, but might also represent a possible trigger initiating the tumor conversion of glial cells in the brain.

## Electronic Supplementary Material

Below is the link to the electronic supplementary material.

ESM 1 (DOCX 28 kb)

ESM 2 (PDF 43 kb)

ESM 3 (PDF 17 kb)
