# Supplementary material for: Inflammation Promotes a Conversion of Astrocytes into Neural Progenitor Cells via NF-κB Activation
Source: Mol Neurobiol. 2015 Sep 17;53(8):5041–55. doi: 10.1007/s12035-015-9428-3 (PMC5012156; doi:10.1007/s12035-015-9428-3)
Supplement: Supplementary file 1 — (DOCX 28 kb) [file 12035_2015_9428_MOESM1_ESM.docx]

**Supplemental data**

**SD1.** Effects of NFκB inhibition on the dedifferentiation of astrocytes. NFκB inhibitor JSH23 was added 30 min prior to 24h of TNF treatment on primary astrocytes. Real-time PCR was used to assess the regulation of *TNF*. Gene expression has been normalized to ß-actin. n = 3, error bars represent the SEM.

**SD2.** Oct4 protein expression in rat C6 glioma cell line. Cells were analyzed by immunofluorescence for the expression of Oct4 (green), Nestin (red) and Dapi (blue). Scale bar = 50 µm.

**SD3.** Oct4 protein expression in mouse neural stem cell. After 2 hours of neurospheres differentiation, cells were analyzed by immunofluorescence for the expression of Oct4 (green), vimentin (red). Scale bar = 50 µm.

**SD4.** Effects of *Oct4* down-regulation on astrocytes dedifferentiation. Primary astrocytes were exposed to siRNA (70 nM) during 6 h and activated with TNF. **(A)** RNA were collected 24 hours, 48 hours, 3 days and 4 days after TNF treatment and analyzed for the expression of *Oct4*, *CD44*, *GFAP*, *Glycogen Phosphorylase* and *PTG* by real-time PCR. Gene expression has been normalized to ß-actin. *, p<0.05 for the two-way Anova main effect of factor “siRNA”; n = 3 independent experiments; error bars represent the SEM.
