# Supplementary figures and images for: Inflammation Promotes a Conversion of Astrocytes into Neural Progenitor Cells via NF-κB Activation
Source: Mol Neurobiol. 2015 Sep 17;53(8):5041–55. doi: 10.1007/s12035-015-9428-3 (PMC5012156; doi:10.1007/s12035-015-9428-3)

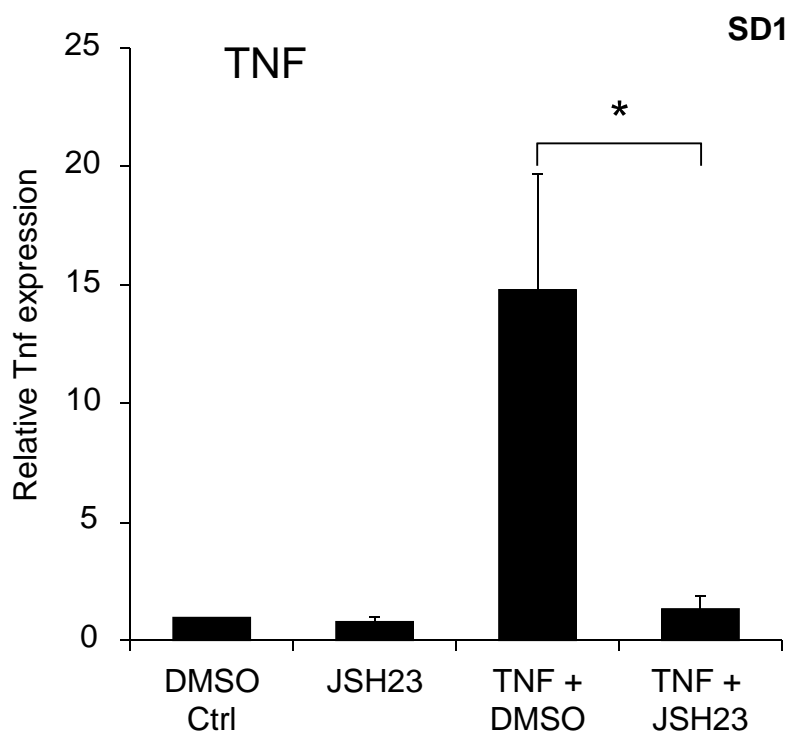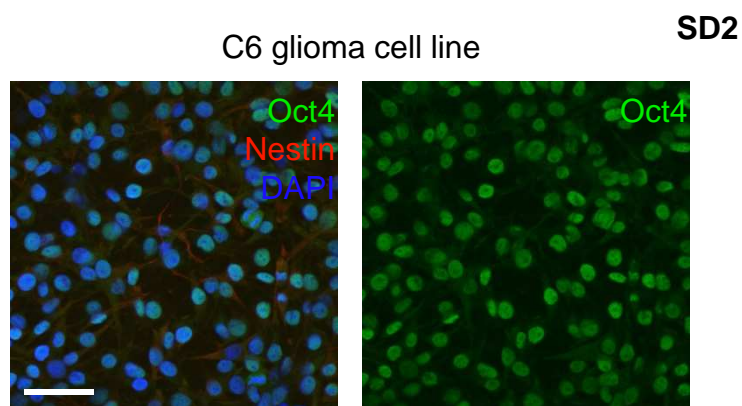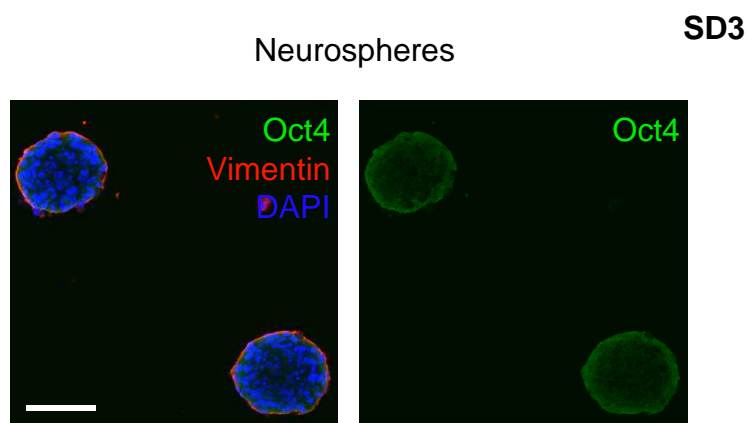

Supplement: Supplementary file 2 — (PDF 43 kb) [file 12035_2015_9428_MOESM2_ESM.pdf]

SD4

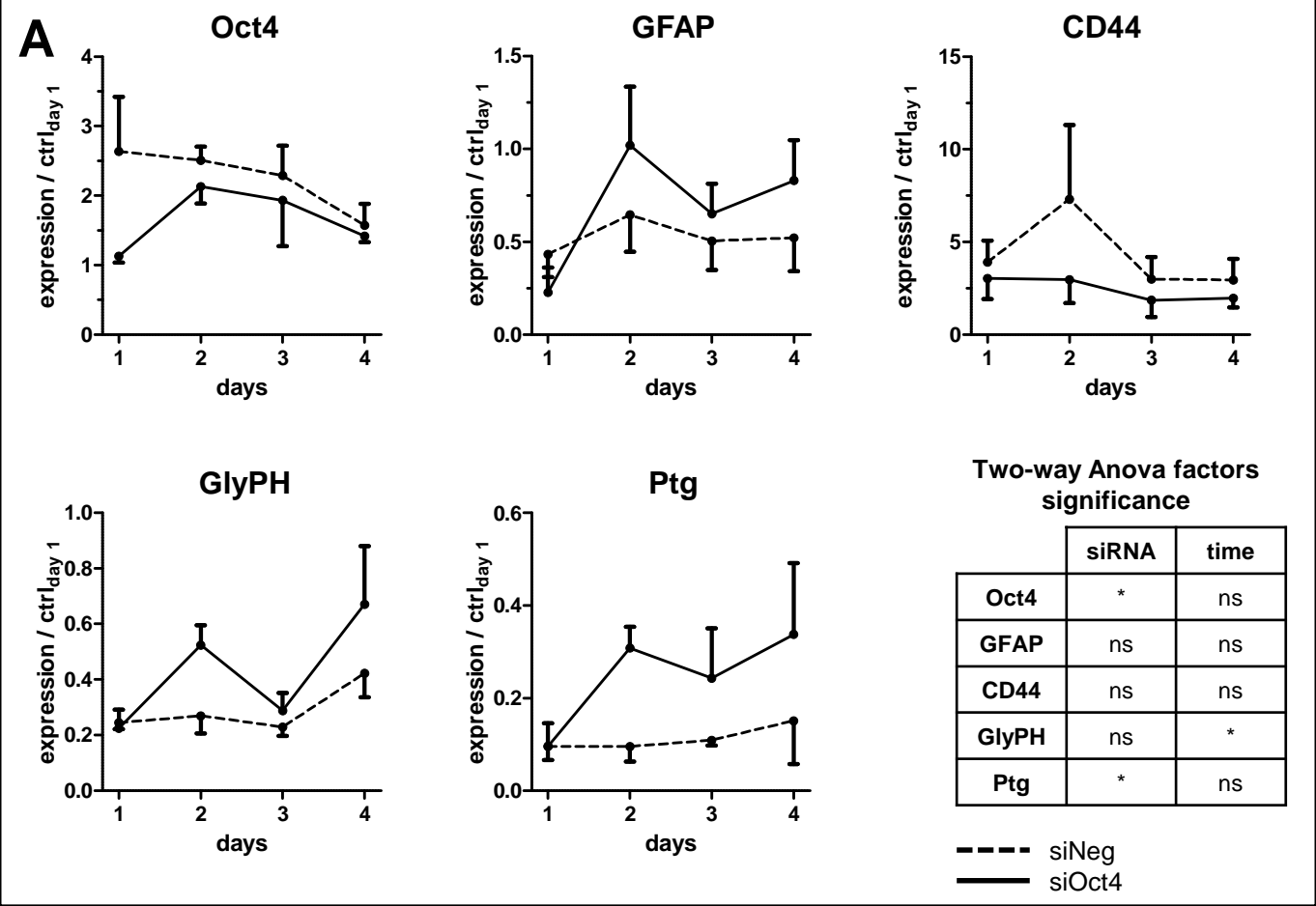

Supplement: Supplementary file 3 — (PDF 17 kb) [file 12035_2015_9428_MOESM3_ESM.pdf]
